# Inhibition of mutagenic translesion synthesis: A possible strategy for improving chemotherapy?

**DOI:** 10.1371/journal.pgen.1006842

**Published:** 2017-08-17

**Authors:** Kinrin Yamanaka, Nimrat Chatterjee, Michael T. Hemann, Graham C. Walker

**Affiliations:** 1 Department of Biology, Massachusetts Institute of Technology, Cambridge, Massachusetts, United States of America; 2 Koch Institute, Massachusetts Institute of Technology, Cambridge, Massachusetts, United States of America; Duke University, UNITED STATES

## Overview

DNA damaging chemotherapy is the first line of treatment for certain cancers, but its long-term success is often marred by the eventual acquisition of chemoresistance. Other cancers cannot be treated because they are intrinsically resistant to such chemotherapy. These 2 types of resistance are coupled in the context of translesion synthesis (TLS), which is carried out by specialized TLS DNA polymerases that can replicate past DNA lesions but in a lower fidelity manner. First, TLS DNA polymerases permit the bypass of modified DNA bases during DNA synthesis, thereby allowing proliferation to continue in the presence of chemotherapy, an issue of particular relevance to intrinsic drug resistance. Second, mistakes introduced by TLS polymerases copying over DNA lesions introduced during the chemotherapy lead to mutations that contribute to acquired resistance. These dual functions of mutagenic TLS polymerases with respect to chemoresistance make these proteins very promising targets for adjuvant therapy. The major branch of mutagenic TLS requires REV1, a Y family DNA polymerase that recruits other TLS polymerases with its C-terminal domain (CTD) including POL ζ, which is also required. Recent evidence obtained using mouse models is summarized, which shows that interfering with REV1/POL ζ-dependent mutagenic TLS during DNA damaging chemotherapy can help overcome problems due to both intrinsic resistance and acquired resistance. Ways to develop drugs that block mutagenic TLS are also considered, including taking advantage of structural knowledge to target key protein-protein interfaces.

## Introduction

While DNA damaging chemotherapy can be very effective and even curative in the treatment of certain cancers, intrinsic and acquired drug resistance underlies tumor progression and morbidity in many cancer patients. Intrinsic resistance defines a cell state that is inherently tolerant of drug action. This can include the activation of drug efflux pumps or detoxifying processes that effectively reduce intracellular drug concentration [1]. This can also include a change in the recognition or persistence of DNA damage, mediated by an enhanced DNA repair capability, a blunted DNA damage response, or the ability to proliferate in the presence of DNA damage. Conversely, acquired drug resistance represents a mutational or epigenetic process by which a chemosensitive cell develops 1 or more of the characteristics of an intrinsically resistant cancer cell. Thus, the mechanisms underlying intrinsic and acquired drug resistance are quite distinct. One describes a cell state, and the other describes the capability of reaching that cell state. Yet, these processes are very much coupled in the context of mutagenic translesion synthesis (TLS).

As discussed throughout this review, mutagenic TLS polymerases underlie 2 important phenotypes in response to genotoxic chemotherapy. First, they allow for the bypass of modified DNA bases during DNA synthesis, allowing proliferation to continue in the presence of chemotherapy. Second, the low fidelity replication performed by TLS polymerases results in the introduction of inappropriate, nonpairing bases across from modified nucleotides. The bypass function of TLS polymerases is particularly relevant to intrinsic drug resistance. Many tumors, including most pancreatic adenocarcinomas, nonsmall cell lung cancers, and aggressive brain tumors, as well as most metastatic malignancies, fail to significantly regress following chemotherapy [2]. In these tumors, TLS activity contributes to a drug resistant state by promoting the tolerance of DNA damage [3–6]. Conversely, the mutational role of TLS polymerases is central to process of acquired drug resistance. Tumor regression and relapse following chemotherapy is almost always accompanied by the development of drug resistant disease. This may not occur at initial relapse, but upon serial cycles of treatment patients generally succumb to tumors that have acquired intrinsically resistant disease. In fact, for certain cancers the overall prognosis is not dictated by the initial response of the tumor to chemotherapy. Rather, the response of the relapsed tumor to therapy is a significantly better determinant of overall survival. For instance, a high error-prone TLS activity translates into greater tumor adaptation to chemotherapy, while a low error-prone TLS activity leaves tumor in a treatment-naïve state. This latter state is amenable to continued long-term treatment of tumors that remain response to treatment with the initial therapy.

The dual functions of mutagenic TLS polymerases in intrinsic and acquired chemoresistance make these proteins very attractive potential targets for adjuvant therapy. When combined with front-line genotoxic therapy, these TLS inhibitors would be expected to sensitize tumors to chemotherapy while blocking drug-induced mutation. Consequently, while the generation of such inhibitors is complex, their route to the clinic is more apparent. TLS inhibitors could be applied in combination with the standard of care for many malignancies. By effectively increasing the effects of chemotherapy in target cells, these agents may also allow for a reduction in chemotherapy dose regimens. An added benefit of these agents may be a reduction in the rate of secondary chemotherapy-driven malignancies that occur in patients following successful treatment of the primary disease.

## TLS polymerases bypass DNA damage

**Fig 1:**
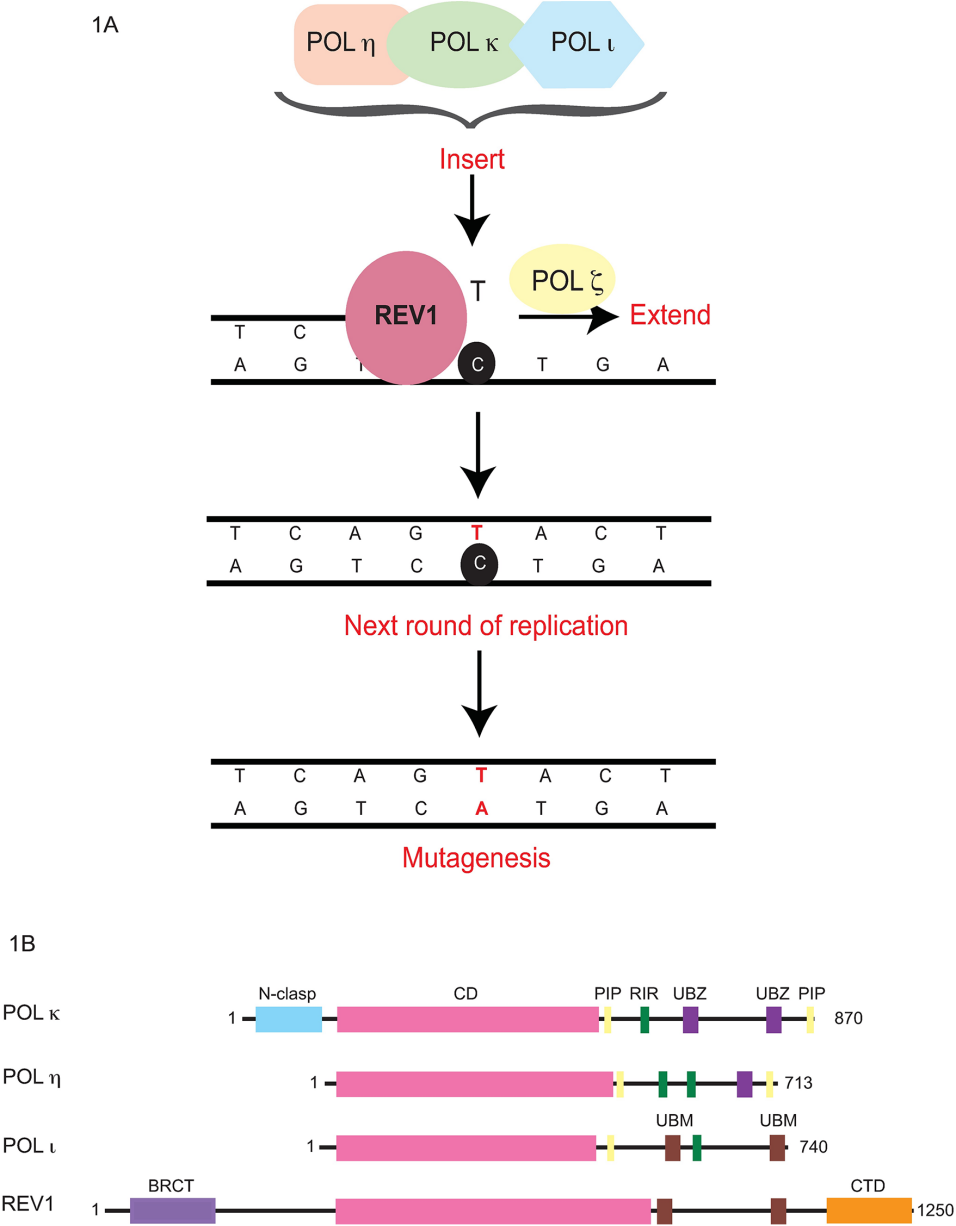
DNA damage bypass process. (A) Mechanism of the 2-step DNA damage bypass process. To bypass DNA damage, REV1 inserts deoxycytidine triphosphates across the damage or orchestrates the recruitment of the other polymerases, POL ι, POL κ, POL η, to replicate across the damage. Thereafter, POL ζ complex can help extend beyond the damage to enable re-initiation of undamaged DNA replication. If an incorrect nucleotide gets incorporated across the damage, this misincorporated nucleotide will lead to a mutation in the next round of replication. (B) A schematic representing the protein domains of the Y-family translesion synthesis (TLS) polymerases, REV1, POL ι, POL κ, POL η.

TLS polymerases are highly conserved, specialized DNA polymerases that can replicate past aberrant DNA lesions but in a lower fidelity manner—a trade-off that preserves genomic integrity in cells [7]. These incorrect nucleotides become fixed into mutations during the next round of DNA replication, contributing to overall fitness and evolution in single cell organisms but propelling tumorigenesis and disease in humans (Fig 1A). There are 10 known human TLS polymerases (REV1, POL η, POL ι, POL κ, POL ζ, POL μ, POL λ, POL β, POL ν, and POL θ), which are distributed in 4 families (Y, B, X, and A), and also Prim Pol, which additionally has primase activity. Although all TLS DNA polymerases are more error-prone than replicative DNA polymerases, some are capable of bypassing specific (cognate) lesions in a relatively error-free manner (Table 1). The extent of DNA synthesis errors during TLS depends on various factors, including the identities of the TLS polymerases employed, the presence or absence of cognate lesions, DNA sequence context, and thermodynamic favorability in the catalytic step [8–10]. The significance of the TLS process to human health is illustrated by xeroderma pigmentosum-variant patients, who are deficient in POL η and are therefore susceptible to UV radiation-induced cancers because the cognate UV-induced cyclobutane pyrimidine dimers are instead bypassed by alternate TLS polymerases (POL ι and POL κ) in a relatively error-prone manner [11, 12].

**Table 1:** Summary of the characteristics, expression, the availability of mouse model, and association to cancers of B- and Y-family translesion synthesis polymerases.

Polymerase	Characteristics	Expression	Mice Model	Cancer Association
REV1 (*REV1)* Y-family	• Exclusively inserts dCMPs opposite template Gs, abasic sites, and adducted G residues [13, 14] • Acts as a scaffolding protein by interacting with both POL ζ and RIR containing POL η, POL κ and POL ι [15, 16] • Generates G/C substitutions during Ig gene somatic hypermutation [17] • Accumulates in DNA damaged induced foci [18–20]	• Protein expression is cytoplasmic in all tissues, with highest in adrenal gland, muscle, liver, etc. (http://www.proteinatlas.org/ENSG00000135945-REV1/tissue) • RNA expressed in all tissues, with highest expression in brain tissues and reproductive organs (http://www.proteinatlas.org/ENSG00000135945-REV1/tissue and https://gtexportal.org/home/gene/REV1)	• *Rev1*^BRCT^ (ΔBRCT region; accelerated skin cancers, genotoxin-induced genome instability) [21, 22]. • *Rev1*^AA^ (defective Rev1 catalytic domain; reduced somatic hypermutation) [23]. • *Rev1*^KO^ (Rev1 deficient; near-infertile and unstable genome) [24]	• Several hepatocarcinomas and occasional lung cancers show high expression of REV1 [25] (http://www.proteinatlas.org/ENSG00000135945-REV1/cancer) • Responsible for drug resistance in ovarian cancer cells [26] • No known somatic mutations in cancers
POL η (*POLH*) Y-family	• Bypasses T-T CPD and cisplatin-GG efficiently, but inefficiently across adducted residues, AP sites, 8-oxo-G [27–32] • Accumulates at DNA damage foci [20, 33]. • Generates A/T substitutions during somatic hypermutagenesis [34]	• Protein expression ubiquitous in nucleus and cytoplasm of all tissues, with high expression in thyroid, lung, pancreas, placenta, testis, etc. (http://www.proteinatlas.org/ENSG00000170734-POLH/tissue) • RNA expressed in all tissues, with highest expression in tonsil, lymph nodes and testis (http://www.proteinatlas.org/ENSG00000170734-POLH/tissue and https://gtexportal.org/home/gene/POLH)	• *Polh*^KO^ (Pol η deficient; fertile, viable, but susceptible to skin cancers, mirrors XP-V phenotype, UV irradiated cells prone to chromatid breaks) [35–37] • *Polh*^+/-^ (slightly susceptible to UV radiation-induced skin carcinogenesis) [35]	• Gene mutations causes XP-V [38] • High expression in single basal cell carcinomas of the skin and some liver cancers (http://www.proteinatlas.org/ENSG00000170734-POLH/cancer) • Enhanced expression in ovarian cancer stem cells [39] • Elevated levels in head and neck tumor samples [40] • 3 missense *POLH* mutations found amongst 201 melanoma patients [41]
POL κ (*POLK*) Y-family	• Propensity to make −1 frameshift mutations, but efficiently bypasses thymine glycols and guanine adducts [42, 43] • Propensity to extend mispaired primer-template termini [44]	• Protein expression data in normal tissues unknown • RNA expressed in all tissues, with slightly high expression in thyroid, parathyroid, endometrium, and testis (http://www.proteinatlas.org/ENSG00000122008-POLK/tissue#gene_information & https://gtexportal.org/home/gene/POLK)	*Polk*^KO^ (Pol κ deficient; fertile, cells are UV sensitive, spontaneous mutator phenotype in kidneys, liver and lungs, and the mice has shortened survival than *Polk*^+/-^ and *Polk*^+/+^ mice) [45, 46]	• Elevated expression in lung cancer [47, 48] • Ectopic overexpression of POL κ induces aneuploidy and carcinogenesis in mice [49] • Two non-coding *POLK* SNPs associated with lung cancer risk [50] • Three somatic *POLK* mutations in 26 prostrate patients [51]
POL ι (*POLI*) Y-family	• Efficiently bypasses template dA; but does so inefficiently on the template dT [52, 53] • Briefly accumulates in replication stress foci [54] • Back-up polymerase in the absence of POL η. Inefficiently bypasses UV damage in the absence of POL η [11, 55]	• High protein expression in parathyroid, thyroid, reproductive organs and pituitary (http://www.proteinatlas.org/ENSG00000101751-POLI/tissue) • High RNA expression in testis, thyroid and parathyroid gland (http://www.proteinatlas.org/ENSG00000101751-POLI/tissue and https://gtexportal.org/home/gene/POLI)	*Poli*^KO^ (Pol ι deficient; mice susceptible to damage-induced lung tumors) [56]. *Polι*^KO^ mice cells not sensitive to DNA damaging agents [57]	• Elevated expression in breast cancer cells [58] • Important candidate for lung neoplasia [59] • Overexpressed in bladder cancer and in esophageal squamous cell carcinoma [60–62] • *POLI* SNP (rs8305) correlated with significant high risk of both lung adenocarcinoma and squamous cell carcinoma [63] • *POLI* SNP (rs3218786) significantly associated with TMPRSS2-ERG fusion-positive prostrate tumors [64]
POL ζ_4_ B-family (REV3 [*REV3*] polymerase, REV7 [*REV7*], POLD2 and POLD3 accessory subunits) [65]	• POL ζ_4_ mediate inefficient TLS across CPDs, (6–4) photoproducts, adducted residues and AP sites, but an error free bypass of thymine glycols [53, 66, 67] • Serves as the key extender polymerase during TLS [68]	• REV3 protein is expressed minimally in the cytoplasm of different tissue types. REV3L transcript is highly expressed in endometrin, smooth muscle, cerebellum and the uterine tissues (http://www.proteinatlas.org/ENSG00000009413-REV3L/tissue and https://gtexportal.org/home/gene/REV3) • High REV7 protein expression in bone marrow and lung tissues. And high REV7 RNA expression in testis, bone marrow, lymph nodes, tonsils, and appendix (http://www.proteinatlas.org/ENSG00000116670-MAD2L2/tissue and https://gtexportal.org/home/gene/MAD2L)	• *Rev3*^KO^ (Rev3 deficient; embryonically lethal and spontaneous and genotoxin induced genome instability) [69–71] • *Rev3*^Δlox^ (conditional Rev3 deficiency; reduced cell proliferation, spontaneous genomic instability and mice develop spontaneously mic lymphoma and spontaneous skin tumors) [72–74] • *Rev7*^KO^ (Rev7 deficient; delayed growth, infertile, reduced cell proliferation, spontaneous genome instability) [75, 76]	• REV7 depletion enhances cisplatin sensitivity in ovarian cancer cells [77] • Loss of REV7 sensitizes ovarian and breast cancer cells to PARP inhibition [78] • High expression in B-cell lymphoma [79] • Elevated expression in colon cancer [80]

AP, apurinic; CPD, cyclobutane pyrimidine dimers; dCMP, deoxycytidine monophosphate; TLS, translesion synthesis; XP-V, xeroderma pigmentosum-variant.

Distinct structural and biochemical features of the TLS polymerases enable them to replicate past the DNA damage. For example, in contrast to classical replicative polymerases, Y-family TLS polymerases possess a smaller thumb and finger domain that makes fewer contacts with DNA and also lack an 3ʹ-5ʹ exonuclease activity to proofread misincorporated nucleotides. Together, these structural attributes result in a larger and/or more permissive catalytic site than replicative polymerases that allows TLS polymerases to accommodate distorted and damaged nucleotides [81, 82]. In addition, other physical features such as the polymerase-associated domain of Y-family polymerases and the wrist and the N-clasp region of POL κ also contribute to polymerase architecture conducive to replication across DNA damage (Fig 1B) [83–87]. Furthermore, regulatory domains of TLS polymerases enable their proper localization and regulation [88]. These special structural features of TLS polymerases are fundamental to their roles in DNA damage bypass.

**Fig 2:**
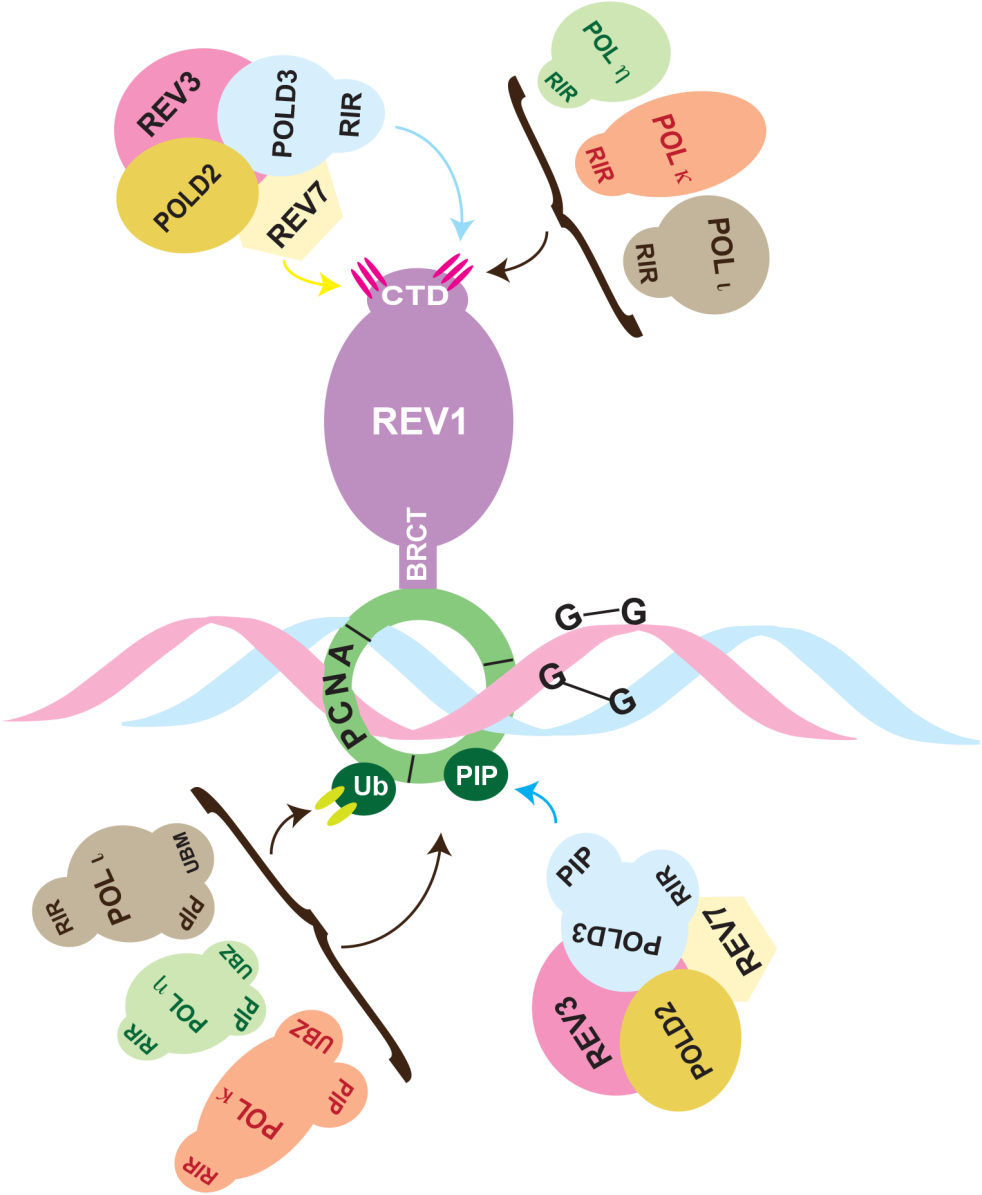
Protein-protein interactions between translesion synthesis (TLS) polymerases are important for the DNA damage bypass process. Two pathways are expected to facilitate TLS across DNA damage—the REV1 dependent and REV1 independent pathway. Majority of the DNA lesions are bypassed in a REV1 dependent fashion, which engages in protein-protein interactions with other TLS polymerases via its C-terminus. REV1 interacts with the REV1-interacting-region (RIR)-containing residues of POL ι, POL κ, POL η to enable insertion of nucleotides across the damage. And REV1 also interacts via key residues with REV7 of the POL ζ complex to facilitate extension beyond the insertion step. REV1 also binds to POLD3 subunit of the POL ζ complex to enable the key switch from the “insertion” to the “extension” step. In the REV1 independent pathway, the RIR-containing polymerases, POL ι, POL κ, POL η, by interacting with the proliferating cell nuclear antigen (PCNA) interacting protein (PIP) and ubiquitin-binding motif (UBM)/ ubiquitin-binding zinc finger (UBZ) domains of PCNA, can also enable TLS at the damaged site. Likewise, the POL ζ complex also interacts with the PIP box of PCNA to access the DNA and enable TLS.

Besides the structural features of individual TLS polymerases, successful TLS also depends on interactions between these polymerases and other cellular proteins that target and choreograph their activity. REV1 functions as a principle scaffolding protein, which recruits other TLS polymerases to first insert a nucleotide opposite the DNA lesion and then eventually help extend the distorted primer-template terminus, in what is recognized as the two-step mechanism of TLS (Fig 2) [7, 8, 89]. For the insertion step, a particular interface of the REV1 CTD interacts with the REV1-interacting-region (RIR) of the inserter polymerases (POL η, POL ι, POL κ). Mutations that disrupt the RIR-interface in the Rev1 CTD prevent interaction with the inserter polymerase in yeast-2 hybrid (Y2H) screens [15, 16, 90, 91]. Insertion across from the damaged base can also be less frequently carried out by REV1 and POL ζ [8]. In the second step, an extender TLS enzyme, a role most frequently fulfilled by POL ζ (REV3/REV7/POLD2/POLD3) and in some cases by POL κ, replaces the inserter and extends the primer-template termini [90]. For the POL ζ -mediated extension step, a different interface in REV1 CTD—distinct from the interface for RIR recognition—makes contact with specific amino acids located on REV7. Mutating residues in the Rev7-interface of the Rev1 CTD inhibits Rev1-Rev7 interaction in Y2H studies and sensitizes chicken DT40 cells to cisplatin [15]. Apart from bypassing DNA damage at stalled replication forks, TLS polymerases also engage in filling single stranded (ss) DNA gaps left behind by replicative polymerases, via the less-well understood gap-filling mechanism [92, 93].

Interestingly, TLS polymerases are also required for other cellular functions. For example, during interstrand cross-link (ICL) repair in replicating cells, certain TLS polymerases—REV1, POL ι, POL κ and POL ν—are required for DNA synthesis over the ICL on the newly exposed leading strand [94–97]. Likewise, in nonreplicating cells, ICL repair depends on the Rev1-POL ζ TLS polymerases to fill the ssDNA-gaps [98]. In a similar fashion, both nucleotide excision repair (NER) and base excision repair (BER) pathways respectively can employ POL κ and POL η to fill the ssDNA gaps left behind after the excising step [99, 100]. Additionally, POL η was recently shown to drive microhomology-mediated break-induced replication (MMBIR) that causes complex genomic rearrangements in yeast and has an important role in homologous recombination (HR) in DT40 cells [101, 102]. Finally, REV1 was recently shown to be required for replication of G-quadruplex structures, thereby influencing epigenetic stability [103]. Independent of its role in TLS, REV7 promotes nonhomologous end joining (NHEJ) at double strand breaks and at telomeres by inhibiting CtIP-mediated end resection [104]. Additionally, REV7 plays a supporting role in cell cycle regulation by sequestering CDH1, which prevents premature activation of the anaphase-promoting complex, thereby inhibiting an exit from mitosis [105]. All these examples are suggestive of an overarching influence of TLS polymerases and their components on cellular physiology, in which they influence DNA damage tolerance, DNA repair, epigenetic stability, and replication across repetitive sequences.

## Modulation of TLS polymerases alters tumor response to chemotherapy

A growing body of evidence now shows that suppression of TLS polymerases not only sensitizes tumor cells to drugs, but also reduces acquisition of drug-induced mutations implicated in tumor resistance. Thus, inhibition of TLS polymerases is a promising new approach to improving cancer therapy. Moreover, in some cancers, TLS polymerases are overexpressed (Table 1),

The impact TLS polymerases have on chemotherapy responses in different cancer subtypes has recently been investigated. In one study, the potential of Rev3 inhibition for the treatment of intrinsically chemoresistant cancers was investigated. A study utilizing the *Kras*^*G12D*^*;p53*^*−/−*^ preclinical model of lung adenocarcinoma showed that, when the level of Rev3 was reduced, these otherwise resistant tumors were sensitized to cisplatin, increasing the overall survival of mice with Rev3-deficient tumors by 2-fold compared with control mice with Rev3-proficient tumors [106]. Reduction of Rev3 or Rev1 in these tumor cells also reduced cisplatin-induced mutagenesis in culture.

**Fig 3:**
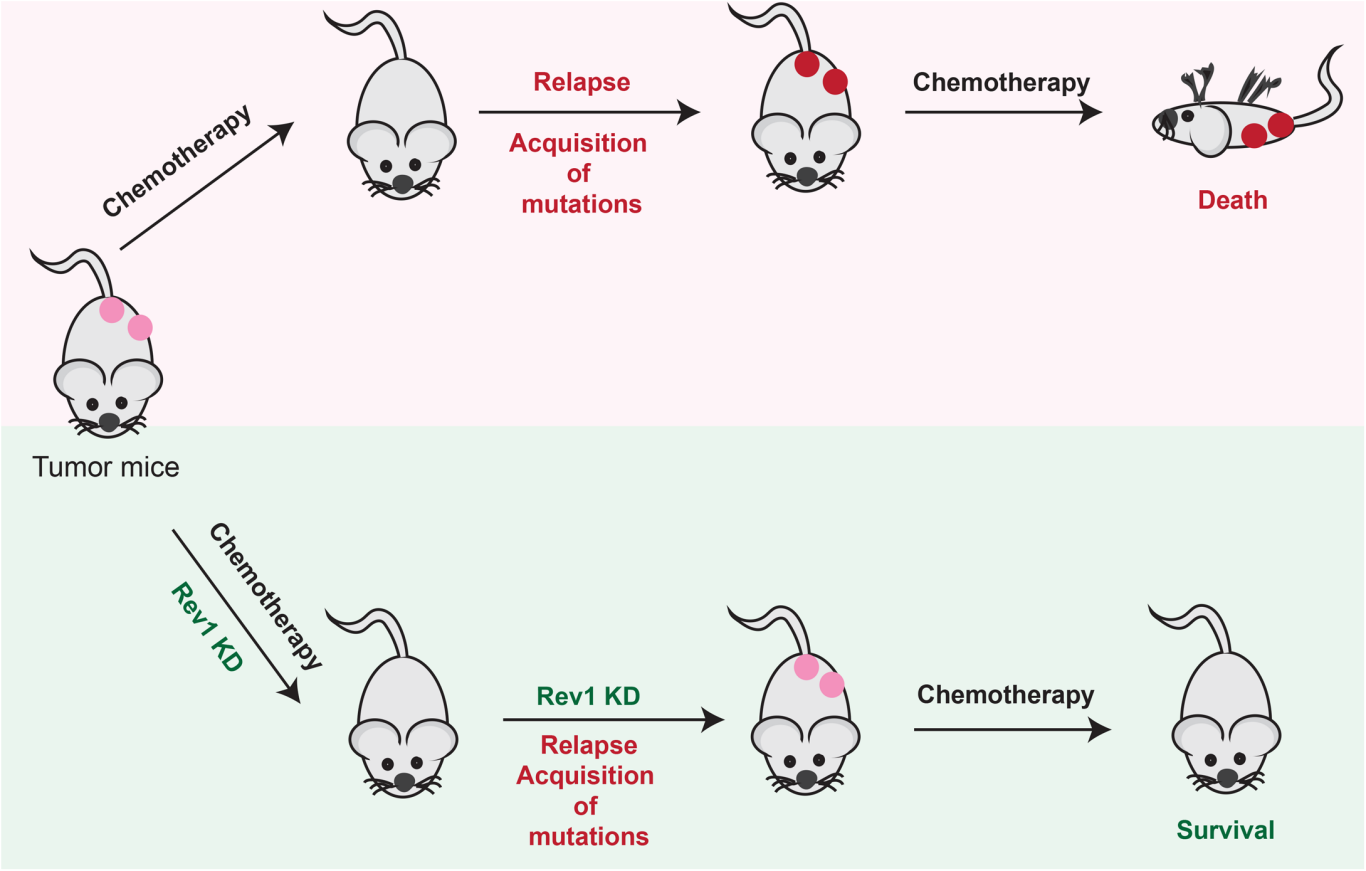
Reduction of Rev1 suppresses chemoresistance. In a tumor mouse model, administration of chemotherapy reduces tumor formation by killing the generally chemoresensitive tumor cells. However, many of the tumors that relapse are resistant to further killing from chemotherapeutic treatment, thereby reducing survival of the mice. In contrast, mice harboring relapsed tumors in which REV1 has been knocked down remain sensitive to chemotherapy, whereby their survival is prolonged.

In a study that employed the *Eμ-myc arf*^-/-^ mouse model of B-cell lymphoma, when mice were subjected to repeated cycles of tumor engraftment and cyclophosphamide treatment, relapsed tumors that appeared after the first round of chemotherapy continued to respond to cyclophosphamide if they were Rev1 deficient. This is in direct contrast to Rev1-proficient relapsed tumors, which exhibited varying degrees of acquired resistance to cyclophosphamide chemotherapy (Fig 3). Additionally, cyclophosphamide-induced mutagenesis of these lymphoma cells in culture was suppressed by Rev1 depletion. These studies showed that Rev1-dependent error-prone bypass of cyclophosphamide-induced DNA damage contributes to the mutagenesis and hence the tumor drug resistance. Thus this study provided the first in vivo evidence that TLS polymerases play a critical role in the development of acquired chemoresistance [107].

Chemotherapy-induced mutagenesis is a phenomenon proposed to cause secondary malignancies and tumor relapse. Hence, targeting REV1 and REV3 might not only increase killing of cancer cells but could also potentially suppress secondary malignancies and tumor relapse. The same principal was explored when an innovative nanoparticle-mediated delivery system was used to target both REV1 and REV3 in combination with a cisplatin prodrug. A nearly complete inhibition of tumor growth and dramatically enhanced survival was observed in LnCaP prostate cancer mouse model [108]. In addition, REV7 depletion has been shown to sensitize ovarian cancer to cisplatin and reduce tumor volumes in nude mice [77]. These studies support the hypothesis that TLS inhibition can suppress at least some classes of intrinsic chemoresistance. Likewise depletion of REV3 in cervical cancer cells [109] or nonsmall cell lung cancer cells [110]; REV1, POL ζ, POL η in HeLa cells [111]; and POL η in ovarian cancer stem cells [39] all sensitize cells to cisplatin. It remains to be seen whether other cancer cell subtypes would similarly respond to knockdown of TLS polymerases and whether observations in cell studies could be recapitulated in mouse models.

Another approach to potentially enhance tumor cell killing via suppression of TLS polymerases is to discover synthetic lethal partners of TLS polymerases. For example, this classical approach is employed in killing BRCA2-deficient tumors by utilizing PARP1 inhibitors [112]. Although a compelling idea, TLS synthetic partners are largely unknown. However, a whole genome siRNA library screen in A549 lung cancer cells identified one gene RRMI—the large subunit of ribonucleotide reductase that confers a synthetic lethal interaction with REV3 [113]. In another lung cancer cell line and in breast cancer cells, ataxia-telangiectasia and Rad3 related inhibition was found to synthetically enhance lethality in cisplatin-treated REV3-deficient cells [114]. In addition, Rev3-deficient DT40 cells exhibited synthetic lethality with RAD54 [115], suggesting a promising potential. Synthetic-lethal partners of TLS polymerases need to be explored in greater detail across other cancer subtypes.

## Drug inhibitors to target TLS polymerases

Taken together, the studies discussed above suggest that small molecules that directly inhibit catalytic functions or disrupt key protein-protein interactions of TLS polymerases could be adjuvants that have the potential to significantly improve chemotherapy. For example, fluorescence-based assays conducted in high-throughput platforms were used to search for small molecule inhibitors that affect catalytic functions of TLS polymerases. Pamoic acid, aurintricarboxylic acid, and ellagic acid were found to inhibit POL ι and POL η [112], while candesartan cilexetil inhibited the enzymatic function of POL κ as well as enhanced UV-induced cytotoxicity in xeroderma pigmentosum-variant (XP-V) cells [116]. Likewise, 3-*O*-methylfunicone, a natural compound isolated from a marine fungal strain, selectively inhibited mammalian Y-family TLS polymerase activity (POL κ, POL, ι, POL η) [117]. Further studies are required to identify compounds with improved specificity and potency.

Very recently small molecules inhibitors that target TLS DNA polymerase protein-protein interactions have been shown to be possible therapeutic candidates. For example, a small molecule inhibitor that binds to REV7 and inhibits its interaction with REV3 was shown to partially suppress ICL repair [118]. Whether the same drug could also suppress TLS is worth investigating. Similarly, detailed structural knowledge of other TLS interfaces, such as between REV1 and REV7 and between REV1 and RIR carrying proteins could be exploited in drug discovery and design.

## Perspective and conclusion

Inhibiting TLS polymerases is a promising approach to improve chemotherapy as it could increase killing of cancer cells, while at the same time reducing the possibility of relapse and acquired drug resistance by reducing chemotherapy-induced mutagenesis. Even cancers known to be intrinsically drug resistant could potentially be sensitized by this approach. Additionally, TLS specific inhibition could also potentially target other repair and recombination pathways that involve TLS polymerases including NER, BER, MMBIR, HR, and NHEJ. However, several outstanding questions still need to be addressed, for example, improving understanding of the structural basis of key protein-protein interactions made by the TLS polymerases. Recently it was shown that the subunits of replicative polymerases cross talk with TLS Polymerases. For instance, the POLD3 subunit of the replicative DNA polymerase POL δ possess an RIR that interacts with the RIR-interface of REV1 CTD, while the POLD2 subunit of POL δ interacts with POL η [90]. These observations suggest that the TLS mechanism is even more complex than previously anticipated and that drug inhibitors for 1 TLS polymerase could potentially target multiple other TLS polymerases. An added complication is that TLS polymerases η, ι, and κ can also function independently of REV1 by interacting with proliferating cell nuclear antigen (PCNA) via the UBM/UBZ domain and the PCNA interacting protein (PIP) domain (Fig 2). It is not known quantitatively what percent of DNA damage in the cells is bypassed in a Rev1-dependent versus REV1-independent manner. This knowledge will help decipher whether a single inhibitor targeting the Rev1/RIR or the REV1/REV7 interaction or a combination of inhibitors targeting the REV1/RIR, REV1/Rev7 and UBM/UBZ-PIP-PCNA interactions would be required for a complete TLS inhibition. Also, a better understanding of synthetic lethal partners of TLS polymerases would provide insights into which tumors might be most susceptible to chemotherapy treatments involving small molecule inhibitors of TLS polymerases. Finally, the effectiveness of small molecule inhibitors of TLS polymerase could be further improved by delivery systems that could target these drugs to specific tumors in cancer patients. Because protein-protein interactions are so important for TLS, drug targets for these interaction interfaces could be promising candidates for cancer therapeutics.

## References

[1] Zahreddine H, Borden KLB. Mechanisms and insights into drug resistance in cancer. Front Pharmacol. 2013;4 doi: 10.3389/fphar.2013.00028 23504227 10.3389/fphar.2013.00028 PMC3596793

[2] Neesse A, Michl P, Frese KK, Feig C, Cook N, Jacobetz MA, et al Stromal biology and therapy in pancreatic cancer. Gut. 2011;60(6):861–8. doi: 10.1136/gut.2010.226092 . 20966025 10.1136/gut.2010.226092

[3] Knobel PA, Marti TM. Translesion DNA synthesis in the context of cancer research. Cancer Cell Int. 2011;11:39 doi: 10.1186/1475-2867-11-39 ; PubMed Central PMCID: PMCPMC3224763. 22047021 10.1186/1475-2867-11-39 PMC3224763

[4] Zhang X, Chen Q, Chen J, He C, Mao J, Dai Y, et al Association of polymorphisms in translesion synthesis genes with prognosis of advanced non-small-cell lung cancer patients treated with platinum-based chemotherapy. J Surg Oncol. 2016;113(1):17–23. doi: 10.1002/jso.24103 . 26611653 10.1002/jso.24103

[5] Wang H, Wu W, Wang HW, Wang S, Chen Y, Zhang X, et al Analysis of specialized DNA polymerases expression in human gliomas: association with prognostic significance. Neuro Oncol. 2010;12(7):679–86. doi: 10.1093/neuonc/nop074 ; PubMed Central PMCID: PMCPMC2940659. 20164241 10.1093/neuonc/nop074 PMC2940659

[6] Broustas CG, Lieberman HB. DNA damage response genes and the development of cancer metastasis. Radiat Res. 2014;181(2):111–30. doi: 10.1667/RR13515.1 ; PubMed Central PMCID: PMCPMC4064942. 24397478 10.1667/RR13515.1 PMC4064942

[7] Sale J.E. LARa RW. Y-family DNA polymerases and their role in tolerance of cellular DNA damage. Nat Rev Mol Cell Biol. 2013;13(3):141–52. 10.1038/nrm3289 PMC3630503 22358330

[8] Waters LS, Minesinger BK, Wiltrout ME, D'Souza S, Woodruff RV, Walker GC. Eukaryotic translesion polymerases and their roles and regulation in DNA damage tolerance. Microbiol Mol Biol Rev. 2009;73(1):134–54. doi: 10.1128/MMBR.00034-08 ; PubMed Central PMCID: PMCPMC2650891. 19258535 10.1128/MMBR.00034-08 PMC2650891

[9] Pages V, Fuchs RP. How DNA lesions are turned into mutations within cells? Oncogene. 2002;21(58):8957–66. doi: 10.1038/sj.onc.1206006 . 12483512 10.1038/sj.onc.1206006

[10] McCulloch SD, Kokoska RJ, Kunkel TA. Efficiency, fidelity and enzymatic switching during translesion DNA synthesis. Cell Cycle. 2004;3(5):580–3. . 15118407

[11] Wang Y, Woodgate R, McManus TP, Mead S, McCormick JJ, Maher VM. Evidence that in xeroderma pigmentosum variant cells, which lack DNA polymerase eta, DNA polymerase iota causes the very high frequency and unique spectrum of UV-induced mutations. Cancer Res. 2007;67(7):3018–26. doi: 10.1158/0008-5472.CAN-06-3073 . 17409408 10.1158/0008-5472.CAN-06-3073

[12] Ziv O, Geacintov N, Nakajima S, Yasui A, Livneh Z. DNA polymerase zeta cooperates with polymerases kappa and iota in translesion DNA synthesis across pyrimidine photodimers in cells from XPV patients. Proc Natl Acad Sci U S A. 2009;106(28):11552–7. doi: 10.1073/pnas.0812548106 ; PubMed Central PMCID: PMCPMC2710681. 19564618 10.1073/pnas.0812548106 PMC2710681

[13] Nelson JR, Lawrence CW, Hinkle DC. Deoxycytidyl transferase activity of yeast REV1 protein. Nature. 1996;382(6593):729–31. doi: 10.1038/382729a0 . 8751446 10.1038/382729a0

[14] Washington MT, Minko IG, Johnson RE, Haracska L, Harris TM, Lloyd RS, et al Efficient and error-free replication past a minor-groove N2-guanine adduct by the sequential action of yeast Rev1 and DNA polymerase zeta. Mol Cell Biol. 2004;24(16):6900–6. doi: 10.1128/MCB.24.16.6900-6906.2004 ; PubMed Central PMCID: PMCPMC479736. 15282292 10.1128/MCB.24.16.6900-6906.2004 PMC479736

[15] Wojtaszek J, Lee CJ, D'Souza S, Minesinger B, Kim H, D'Andrea AD, et al Structural basis of Rev1-mediated assembly of a quaternary vertebrate translesion polymerase complex consisting of Rev1, heterodimeric polymerase (Pol) zeta, and Pol kappa. J Biol Chem. 2012;287(40):33836–46. doi: 10.1074/jbc.M112.394841 ; PubMed Central PMCID: PMCPMC3460478. 22859295 10.1074/jbc.M112.394841 PMC3460478

[16] Wojtaszek J, Liu J, D'Souza S, Wang S, Xue Y, Walker GC, et al Multifaceted recognition of vertebrate Rev1 by translesion polymerases zeta and kappa. J Biol Chem. 2012;287(31):26400–8. doi: 10.1074/jbc.M112.380998 ; PubMed Central PMCID: PMCPMC3406723. 22700975 10.1074/jbc.M112.380998 PMC3406723

[17] Simpson LJ, Sale JE. Rev1 is essential for DNA damage tolerance and non-templated immunoglobulin gene mutation in a vertebrate cell line. EMBO J. 2003;22(7):1654–64. doi: 10.1093/emboj/cdg161 ; PubMed Central PMCID: PMCPMC152905. 12660171 10.1093/emboj/cdg161 PMC152905

[18] Mukhopadhyay S, Clark DR, Watson NB, Zacharias W, McGregor WG. REV1 accumulates in DNA damage-induced nuclear foci in human cells and is implicated in mutagenesis by benzo[a]pyrenediolepoxide. Nucleic Acids Res. 2004;32(19):5820–6. doi: 10.1093/nar/gkh903 ; PubMed Central PMCID: PMCPMC528789. 15523096 10.1093/nar/gkh903 PMC528789

[19] Kim H, Yang K, Dejsuphong D, D'Andrea AD. Regulation of Rev1 by the Fanconi anemia core complex. Nat Struct Mol Biol. 2012;19(2):164–70. doi: 10.1038/nsmb.2222 ; PubMed Central PMCID: PMCPMC3280818. 22266823 10.1038/nsmb.2222 PMC3280818

[20] Tissier A, Kannouche P, Reck MP, Lehmann AR, Fuchs RP, Cordonnier A. Co-localization in replication foci and interaction of human Y-family members, DNA polymerase pol eta and REVl protein. DNA Repair (Amst). 2004;3(11):1503–14. doi: 10.1016/j.dnarep.2004.06.015 . 15380106 10.1016/j.dnarep.2004.06.015

[21] Jansen JG, Tsaalbi-Shtylik A, Langerak P, Calleja F, Meijers CM, Jacobs H, et al The BRCT domain of mammalian Rev1 is involved in regulating DNA translesion synthesis. Nucleic Acids Res. 2005;33(1):356–65. doi: 10.1093/nar/gki189 ; PubMed Central PMCID: PMCPMC546167. 15653636 10.1093/nar/gki189 PMC546167

[22] Tsaalbi-Shtylik A, Verspuy JW, Jansen JG, Rebel H, Carlee LM, van der Valk MA, et al Error-prone translesion replication of damaged DNA suppresses skin carcinogenesis by controlling inflammatory hyperplasia. Proc Natl Acad Sci U S A. 2009;106(51):21836–41. doi: 10.1073/pnas.0909507106 ; PubMed Central PMCID: PMCPMC2799833. 20007784 10.1073/pnas.0909507106 PMC2799833

[23] Masuda K, Ouchida R, Li Y, Gao X, Mori H, Wang JY. A critical role for REV1 in regulating the induction of C:G transitions and A:T mutations during Ig gene hypermutation. J Immunol. 2009;183(3):1846–50. doi: 10.4049/jimmunol.0901240 . 19587019 10.4049/jimmunol.0901240

[24] Krijger PH, Tsaalbi-Shtylik A, Wit N, van den Berk PC, de Wind N, Jacobs H. Rev1 is essential in generating G to C transversions downstream of the Ung2 pathway but not the Msh2+Ung2 hybrid pathway. Eur J Immunol. 2013;43(10):2765–70. doi: 10.1002/eji.201243191 . 23857323 10.1002/eji.201243191

[25] Dumstorf CA, Mukhopadhyay S, Krishnan E, Haribabu B, McGregor WG. REV1 is implicated in the development of carcinogen-induced lung cancer. Mol Cancer Res. 2009;7(2):247–54. doi: 10.1158/1541-7786.MCR-08-0399 ; PubMed Central PMCID: PMCPMC2644734. 19176310 10.1158/1541-7786.MCR-08-0399 PMC2644734

[26] Lin X, Okuda T, Trang J, Howell SB. Human REV1 modulates the cytotoxicity and mutagenicity of cisplatin in human ovarian carcinoma cells. Mol Pharmacol. 2006;69(5):1748–54. doi: 10.1124/mol.105.020446 . 16495473 10.1124/mol.105.020446

[27] Klarer AC, Stallons LJ, Burke TJ, Skaggs RL, McGregor WG. DNA polymerase eta participates in the mutagenic bypass of adducts induced by benzo[a]pyrene diol epoxide in mammalian cells. PLoS ONE. 2012;7(6):e39596 doi: 10.1371/journal.pone.0039596 ; PubMed Central PMCID: PMCPMC3380003. 22745795 10.1371/journal.pone.0039596 PMC3380003

[28] Stary A, Kannouche P, Lehmann AR, Sarasin A. Role of DNA polymerase eta in the UV mutation spectrum in human cells. J Biol Chem. 2003;278(21):18767–75. doi: 10.1074/jbc.M211838200 . 12644471 10.1074/jbc.M211838200

[29] Maga G, Villani G, Crespan E, Wimmer U, Ferrari E, Bertocci B, et al 8-oxo-guanine bypass by human DNA polymerases in the presence of auxiliary proteins. Nature. 2007;447(7144):606–8. doi: 10.1038/nature05843 . 17507928 10.1038/nature05843

[30] Hendel A, Ziv O, Gueranger Q, Geacintov N, Livneh Z. Reduced efficiency and increased mutagenicity of translesion DNA synthesis across a TT cyclobutane pyrimidine dimer, but not a TT 6–4 photoproduct, in human cells lacking DNA polymerase eta. DNA Repair (Amst). 2008;7(10):1636–46. doi: 10.1016/j.dnarep.2008.06.008 ; PubMed Central PMCID: PMCPMC2656611. 18634905 10.1016/j.dnarep.2008.06.008 PMC2656611

[31] Masutani C, Kusumoto R, Iwai S, Hanaoka F. Mechanisms of accurate translesion synthesis by human DNA polymerase eta. EMBO J. 2000;19(12):3100–9. doi: 10.1093/emboj/19.12.3100 ; PubMed Central PMCID: PMCPMC203367. 10856253 10.1093/emboj/19.12.3100 PMC203367

[32] Zhao Y, Biertumpfel C, Gregory MT, Hua YJ, Hanaoka F, Yang W. Structural basis of human DNA polymerase eta-mediated chemoresistance to cisplatin. Proc Natl Acad Sci U S A. 2012;109(19):7269–74. doi: 10.1073/pnas.1202681109 ; PubMed Central PMCID: PMCPMC3358888. 22529383 10.1073/pnas.1202681109 PMC3358888

[33] Solovjeva L, Svetlova M, Sasina L, Tanaka K, Saijo M, Nazarov I, et al High mobility of flap endonuclease 1 and DNA polymerase eta associated with replication foci in mammalian S-phase nucleus. Mol Biol Cell. 2005;16(5):2518–28. doi: 10.1091/mbc.E04-12-1066 ; PubMed Central PMCID: PMCPMC1087254. 15758026 10.1091/mbc.E04-12-1066 PMC1087254

[34] Zeng X, Winter DB, Kasmer C, Kraemer KH, Lehmann AR, Gearhart PJ. DNA polymerase eta is an A-T mutator in somatic hypermutation of immunoglobulin variable genes. Nat Immunol. 2001;2(6):537–41. doi: 10.1038/88740 . 11376341 10.1038/88740

[35] Lin Q, Clark AB, McCulloch SD, Yuan T, Bronson RT, Kunkel TA, et al Increased susceptibility to UV-induced skin carcinogenesis in polymerase eta-deficient mice. Cancer Res. 2006;66(1):87–94. doi: 10.1158/0008-5472.CAN-05-1862 . 16397220 10.1158/0008-5472.CAN-05-1862

[36] Rey L, Sidorova JM, Puget N, Boudsocq F, Biard DS, Monnat RJ Jr, et al Human DNA polymerase eta is required for common fragile site stability during unperturbed DNA replication. Mol Cell Biol. 2009;29(12):3344–54. doi: 10.1128/MCB.00115-09 ; PubMed Central PMCID: PMCPMC2698728. 19380493 10.1128/MCB.00115-09 PMC2698728

[37] Ito W, Yokoi M, Sakayoshi N, Sakurai Y, Akagi J, Mitani H, et al Stalled Poleta at its cognate substrate initiates an alternative translesion synthesis pathway via interaction with REV1. Genes Cells. 2012;17(2):98–108. doi: 10.1111/j.1365-2443.2011.01576.x . 22244149 10.1111/j.1365-2443.2011.01576.x

[38] Masutani C, Kusumoto R, Yamada A, Dohmae N, Yokoi M, Yuasa M, et al The XPV (xeroderma pigmentosum variant) gene encodes human DNA polymerase eta. Nature. 1999;399(6737):700–4. doi: 10.1038/21447 . 10385124 10.1038/21447

[39] Srivastava AK, Han C, Zhao R, Cui T, Dai Y, Mao C, et al Enhanced expression of DNA polymerase eta contributes to cisplatin resistance of ovarian cancer stem cells. Proc Natl Acad Sci U S A. 2015;112(14):4411–6. Epub 2015/04/02. doi: 10.1073/pnas.1421365112 ; PubMed Central PMCID: PMC4394248. 25831546 10.1073/pnas.1421365112 PMC4394248

[40] Zhou W, Chen YW, Liu X, Chu P, Loria S, Wang Y, et al Expression of DNA translesion synthesis polymerase eta in head and neck squamous cell cancer predicts resistance to gemcitabine and cisplatin-based chemotherapy. PLoS ONE. 2013;8(12):e83978 doi: 10.1371/journal.pone.0083978 ; PubMed Central PMCID: PMCPMC3869838. 24376779 10.1371/journal.pone.0083978 PMC3869838

[41] Di Lucca J, Guedj M, Lacapere JJ, Fargnoli MC, Bourillon A, Dieude P, et al Variants of the xeroderma pigmentosum variant gene (POLH) are associated with melanoma risk. Eur J Cancer. 2009;45(18):3228–36. doi: 10.1016/j.ejca.2009.04.034 . 19477635 10.1016/j.ejca.2009.04.034

[42] Ogi T, Kato T Jr., Kato T, Ohmori H. Mutation enhancement by DINB1, a mammalian homologue of the Escherichia coli mutagenesis protein dinB. Genes Cells. 1999;4(11):607–18. . 10620008 10.1046/j.1365-2443.1999.00289.x

[43] Choi JY, Angel KC, Guengerich FP. Translesion synthesis across bulky N2-alkyl guanine DNA adducts by human DNA polymerase kappa. J Biol Chem. 2006;281(30):21062–72. doi: 10.1074/jbc.M602246200 . 16751196 10.1074/jbc.M602246200

[44] Washington MT, Johnson RE, Prakash L, Prakash S. Human DINB1-encoded DNA polymerase kappa is a promiscuous extender of mispaired primer termini. Proc Natl Acad Sci U S A. 2002;99(4):1910–4. doi: 10.1073/pnas.032594399 ; PubMed Central PMCID: PMCPMC122293. 11842189 10.1073/pnas.032594399 PMC122293

[45] Schenten D, Gerlach VL, Guo C, Velasco-Miguel S, Hladik CL, White CL, et al DNA polymerase kappa deficiency does not affect somatic hypermutation in mice. Eur J Immunol. 2002;32(11):3152–60. doi: 10.1002/1521-4141(200211)32:11<3152::AID-IMMU3152>3.0.CO;2-2 . 12555660 10.1002/1521-4141(200211)32:11<3152::AID-IMMU3152>3.0.CO;2-2

[46] Stancel JN, McDaniel LD, Velasco S, Richardson J, Guo C, Friedberg EC. Polk mutant mice have a spontaneous mutator phenotype. DNA Repair (Amst). 2009;8(12):1355–62. doi: 10.1016/j.dnarep.2009.09.003 ; PubMed Central PMCID: PMCPMC2787749. 19783230 10.1016/j.dnarep.2009.09.003 PMC2787749

[47] Wang Y, Seimiya M, Kawamura K, Yu L, Ogi T, Takenaga K, et al Elevated expression of DNA polymerase kappa in human lung cancer is associated with p53 inactivation: Negative regulation of POLK promoter activity by p53. Int J Oncol. 2004;25(1):161–5. . 15202001

[48] J OW, Kawamura K, Tada Y, Ohmori H, Kimura H, Sakiyama S, et al DNA polymerase kappa, implicated in spontaneous and DNA damage-induced mutagenesis, is overexpressed in lung cancer. Cancer Res. 2001;61(14):5366–9. . 11454676

[49] Bavoux C, Leopoldino AM, Bergoglio V, J OW, Ogi T, Bieth A, et al Up-regulation of the error-prone DNA polymerase {kappa} promotes pleiotropic genetic alterations and tumorigenesis. Cancer Res. 2005;65(1):325–30. . 15665310

[50] Michiels S, Danoy P, Dessen P, Bera A, Boulet T, Bouchardy C, et al Polymorphism discovery in 62 DNA repair genes and haplotype associations with risks for lung and head and neck cancers. Carcinogenesis. 2007;28(8):1731–9. doi: 10.1093/carcin/bgm111 . 17494052 10.1093/carcin/bgm111

[51] Makridakis NM, Phipps T, Srivastav S, Reichardt JK. PCR-free method detects high frequency of genomic instability in prostate cancer. Nucleic Acids Res. 2009;37(22):7441–6. doi: 10.1093/nar/gkp761 ; PubMed Central PMCID: PMCPMC2794161. 19797393 10.1093/nar/gkp761 PMC2794161

[52] Choi JY, Lim S, Eoff RL, Guengerich FP. Kinetic analysis of base-pairing preference for nucleotide incorporation opposite template pyrimidines by human DNA polymerase iota. J Mol Biol. 2009;389(2):264–74. doi: 10.1016/j.jmb.2009.04.023 ; PubMed Central PMCID: PMCPMC4010588. 19376129 10.1016/j.jmb.2009.04.023 PMC4010588

[53] Johnson RE, Washington MT, Haracska L, Prakash S, Prakash L. Eukaryotic polymerases iota and zeta act sequentially to bypass DNA lesions. Nature. 2000;406(6799):1015–9. doi: 10.1038/35023030 . 10984059 10.1038/35023030

[54] Kannouche P, Fernandez de Henestrosa AR, Coull B, Vidal AE, Gray C, Zicha D, et al Localization of DNA polymerases eta and iota to the replication machinery is tightly co-ordinated in human cells. EMBO J. 2002;21(22):6246–56. ; PubMed Central PMCID: PMCPMC137208. doi: 10.1093/emboj/cdf618 12426396 10.1093/emboj/cdf618 PMC137208

[55] Dumstorf CA, Clark AB, Lin Q, Kissling GE, Yuan T, Kucherlapati R, et al Participation of mouse DNA polymerase iota in strand-biased mutagenic bypass of UV photoproducts and suppression of skin cancer. Proc Natl Acad Sci U S A. 2006;103(48):18083–8. doi: 10.1073/pnas.0605247103 ; PubMed Central PMCID: PMCPMC1838710. 17114294 10.1073/pnas.0605247103 PMC1838710

[56] Lee GH, Matsushita H. Genetic linkage between Pol iota deficiency and increased susceptibility to lung tumors in mice. Cancer Sci. 2005;96(5):256–9. doi: 10.1111/j.1349-7006.2005.00042.x . 15904465 10.1111/j.1349-7006.2005.00042.x PMC11158430

[57] Ohkumo T, Kondo Y, Yokoi M, Tsukamoto T, Yamada A, Sugimoto T, et al UV-B radiation induces epithelial tumors in mice lacking DNA polymerase eta and mesenchymal tumors in mice deficient for DNA polymerase iota. Mol Cell Biol. 2006;26(20):7696–706. doi: 10.1128/MCB.01076-06 ; PubMed Central PMCID: PMCPMC1636855. 17015482 10.1128/MCB.01076-06 PMC1636855

[58] Yang J, Chen Z, Liu Y, Hickey RJ, Malkas LH. Altered DNA polymerase iota expression in breast cancer cells leads to a reduction in DNA replication fidelity and a higher rate of mutagenesis. Cancer Res. 2004;64(16):5597–607. doi: 10.1158/0008-5472.CAN-04-0603 . 15313897 10.1158/0008-5472.CAN-04-0603

[59] Wang M, Devereux TR, Vikis HG, McCulloch SD, Holliday W, Anna C, et al Pol iota is a candidate for the mouse pulmonary adenoma resistance 2 locus, a major modifier of chemically induced lung neoplasia. Cancer Res. 2004;64(6):1924–31. . 15026325 10.1158/0008-5472.can-03-3080

[60] Yuan F, Xu Z, Yang M, Wei Q, Zhang Y, Yu J, et al Overexpressed DNA polymerase iota regulated by JNK/c-Jun contributes to hypermutagenesis in bladder cancer. PLoS ONE. 2013;8(7):e69317 doi: 10.1371/journal.pone.0069317 ; PubMed Central PMCID: PMCPMC3724822. 23922701 10.1371/journal.pone.0069317 PMC3724822

[61] Zhou J, Zhang S, Xie L, Liu P, Xie F, Wu J, et al Overexpression of DNA polymerase iota (Poliota) in esophageal squamous cell carcinoma. Cancer Sci. 2012;103(8):1574–9. doi: 10.1111/j.1349-7006.2012.02309.x . 22509890 10.1111/j.1349-7006.2012.02309.x PMC7659357

[62] Zou S, Shang ZF, Liu B, Zhang S, Wu J, Huang M, et al DNA polymerase iota (Pol iota) promotes invasion and metastasis of esophageal squamous cell carcinoma. Oncotarget. 2016;7(22):32274–85. doi: 10.18632/oncotarget.8580 ; PubMed Central PMCID: PMCPMC5078012. 27057634 10.18632/oncotarget.8580 PMC5078012

[63] Sakiyama T, Kohno T, Mimaki S, Ohta T, Yanagitani N, Sobue T, et al Association of amino acid substitution polymorphisms in DNA repair genes TP53, POLI, REV1 and LIG4 with lung cancer risk. Int J Cancer. 2005;114(5):730–7. doi: 10.1002/ijc.20790 . 15609317 10.1002/ijc.20790

[64] Luedeke M, Linnert CM, Hofer MD, Surowy HM, Rinckleb AE, Hoegel J, et al Predisposition for TMPRSS2-ERG fusion in prostate cancer by variants in DNA repair genes. Cancer Epidemiol Biomarkers Prev. 2009;18(11):3030–5. doi: 10.1158/1055-9965.EPI-09-0772 . 19861517 10.1158/1055-9965.EPI-09-0772

[65] Makarova AV, Stodola JL, Burgers PM. A four-subunit DNA polymerase zeta complex containing Pol delta accessory subunits is essential for PCNA-mediated mutagenesis. Nucleic Acids Res. 2012;40(22):11618–26. doi: 10.1093/nar/gks948 ; PubMed Central PMCID: PMCPMC3526297. 23066099 10.1093/nar/gks948 PMC3526297

[66] Gibbs PE, McDonald J, Woodgate R, Lawrence CW. The relative roles in vivo of Saccharomyces cerevisiae Pol eta, Pol zeta, Rev1 protein and Pol32 in the bypass and mutation induction of an abasic site, T-T (6–4) photoadduct and T-T cis-syn cyclobutane dimer. Genetics. 2005;169(2):575–82. doi: 10.1534/genetics.104.034611 ; PubMed Central PMCID: PMCPMC1449107. 15520252 10.1534/genetics.104.034611 PMC1449107

[67] Johnson RE, Yu SL, Prakash S, Prakash L. Yeast DNA polymerase zeta (zeta) is essential for error-free replication past thymine glycol. Genes Dev. 2003;17(1):77–87. doi: 10.1101/gad.1048303 ; PubMed Central PMCID: PMCPMC195962. 12514101 10.1101/gad.1048303 PMC195962

[68] Masuda Y HFa CM. Translesion DNA synthesis and damage tolerance pathways. Sugasawa FHaK, editor. Japan: Springer; 2016.

[69] J OW, Kajiwara K, Kawamura K, Kimura M, Miyagishima H, Koseki H, et al An essential role for REV3 in mammalian cell survival: absence of REV3 induces p53-independent embryonic death. Biochem Biophys Res Commun. 2002;293(3):1132–7. doi: 10.1016/S0006-291X(02)00341-8 . 12051777 10.1016/S0006-291X(02)00341-8

[70] Van Sloun PP, Varlet I, Sonneveld E, Boei JJ, Romeijn RJ, Eeken JC, et al Involvement of mouse Rev3 in tolerance of endogenous and exogenous DNA damage. Mol Cell Biol. 2002;22(7):2159–69. ; PubMed Central PMCID: PMCPMC133679. doi: 10.1128/MCB.22.7.2159-2169.2002 11884603 10.1128/MCB.22.7.2159-2169.2002 PMC133679

[71] Wittschieben J, Shivji MK, Lalani E, Jacobs MA, Marini F, Gearhart PJ, et al Disruption of the developmentally regulated Rev3l gene causes embryonic lethality. Curr Biol. 2000;10(19):1217–20. . 11050392 10.1016/s0960-9822(00)00725-9

[72] Bemark M, Khamlichi AA, Davies SL, Neuberger MS. Disruption of mouse polymerase zeta (Rev3) leads to embryonic lethality and impairs blastocyst development in vitro. Curr Biol. 2000;10(19):1213–6. . 11050391 10.1016/s0960-9822(00)00724-7

[73] Lange SS, Bedford E, Reh S, Wittschieben JP, Carbajal S, Kusewitt DF, et al Dual role for mammalian DNA polymerase zeta in maintaining genome stability and proliferative responses. Proc Natl Acad Sci U S A. 2013;110(8):E687–96. doi: 10.1073/pnas.1217425110 ; PubMed Central PMCID: PMCPMC3581960. 23386725 10.1073/pnas.1217425110 PMC3581960

[74] Lange SS, Wittschieben JP, Wood RD. DNA polymerase zeta is required for proliferation of normal mammalian cells. Nucleic Acids Res. 2012;40(10):4473–82. doi: 10.1093/nar/gks054 ; PubMed Central PMCID: PMCPMC3378892. 22319213 10.1093/nar/gks054 PMC3378892

[75] Watanabe N, Mii S, Asai N, Asai M, Niimi K, Ushida K, et al The REV7 subunit of DNA polymerase zeta is essential for primordial germ cell maintenance in the mouse. J Biol Chem. 2013;288(15):10459–71. doi: 10.1074/jbc.M112.421966 ; PubMed Central PMCID: PMCPMC3624428. 23463509 10.1074/jbc.M112.421966 PMC3624428

[76] Jansen JG, Langerak P, Tsaalbi-Shtylik A, van den Berk P, Jacobs H, de Wind N. Strand-biased defect in C/G transversions in hypermutating immunoglobulin genes in Rev1-deficient mice. J Exp Med. 2006;203(2):319–23. doi: 10.1084/jem.20052227 ; PubMed Central PMCID: PMCPMC2118202. 16476771 10.1084/jem.20052227 PMC2118202

[77] Niimi K, Murakumo Y, Watanabe N, Kato T, Mii S, Enomoto A, et al Suppression of REV7 enhances cisplatin sensitivity in ovarian clear cell carcinoma cells. Cancer Sci. 2014;105(5):545–52. doi: 10.1111/cas.12390 ; PubMed Central PMCID: PMCPMC4317831. 24597627 10.1111/cas.12390 PMC4317831

[78] Xu G, Chapman JR, Brandsma I, Yuan J, Mistrik M, Bouwman P, et al REV7 counteracts DNA double-strand break resection and affects PARP inhibition. Nature. 2015;521(7553):541–4. doi: 10.1038/nature14328 ; PubMed Central PMCID: PMCPMC4671316. 25799992 10.1038/nature14328 PMC4671316

[79] Okina S, Yanagisawa N, Yokoyama M, Sakurai Y, Numata Y, Umezawa A, et al High expression of REV7 is an independent prognostic indicator in patients with diffuse large B-cell lymphoma treated with rituximab. Int J Hematol. 2015;102(6):662–9. doi: 10.1007/s12185-015-1880-3 . 26449786 10.1007/s12185-015-1880-3

[80] Rimkus C, Friederichs J, Rosenberg R, Holzmann B, Siewert JR, Janssen KP. Expression of the mitotic checkpoint gene MAD2L2 has prognostic significance in colon cancer. Int J Cancer. 2007;120(1):207–11. doi: 10.1002/ijc.22155 . 17044027 10.1002/ijc.22155

[81] Yang W. Damage repair DNA polymerases Y. Curr Opin Struct Biol. 2003;13(1):23–30. . 12581656 10.1016/s0959-440x(02)00003-9

[82] Yang W. Potraits of a Y-family DNA polymerase. FEBS Lett. 2005;579:868–72. doi: 10.1016/j.febslet.2004.11.047 15680965 10.1016/j.febslet.2004.11.047

[83] Ling H, Boudsocq F, Plosky BS, Woodgate R, Yang W. Replication of a cis-syn thymine dimer at atomic resolution. Nature. 2003;424(6952):1083–7. doi: 10.1038/nature01919 . 12904819 10.1038/nature01919

[84] Lone S, Townson SA, Uljon SN, Johnson RE, Brahma A, Nair DT, et al Human DNA polymerase kappa encircles DNA: implications for mismatch extension and lesion bypass. Mol Cell. 2007;25(4):601–14. doi: 10.1016/j.molcel.2007.01.018 . 17317631 10.1016/j.molcel.2007.01.018

[85] Trincao J, Johnson RE, Escalante CR, Prakash S, Prakash L, Aggarwal AK. Structure of the catalytic core of S. cerevisiae DNA polymerase eta: implications for translesion DNA synthesis. Mol Cell. 2001;8(2):417–26. . 11545743 10.1016/s1097-2765(01)00306-9

[86] Jansen JG, Tsaalbi-Shtylik A, Hendriks G, Verspuy J, Gali H, Haracska L, et al Mammalian polymerase zeta is essential for post-replication repair of UV-induced DNA lesions. DNA Repair (Amst). 2009;8(12):1444–51. doi: 10.1016/j.dnarep.2009.09.006 . 19783229 10.1016/j.dnarep.2009.09.006

[87] Jansen JG, Tsaalbi-Shtylik A, Hendriks G, Gali H, Hendel A, Johansson F, et al Separate domains of Rev1 mediate two modes of DNA damage bypass in mammalian cells. Mol Cell Biol. 2009;29(11):3113–23. doi: 10.1128/MCB.00071-09 ; PubMed Central PMCID: PMCPMC2682010. 19332561 10.1128/MCB.00071-09 PMC2682010

[88] Goodman MF, Woodgate R. Translesion DNA polymerases. Cold Spring Harb Perspect Biol. 2013;5(10):a010363 doi: 10.1101/cshperspect.a010363 ; PubMed Central PMCID: PMCPMC3783050. 23838442 10.1101/cshperspect.a010363 PMC3783050

[89] Yang W, Woodgate R. What a difference a decade makes: insights into translesion DNA synthesis. Proc Natl Acad Sci U S A. 2007;104(40):15591–8. doi: 10.1073/pnas.0704219104 ; PubMed Central PMCID: PMCPMC2000391. 17898175 10.1073/pnas.0704219104 PMC2000391

[90] Pustovalova Y, Magalhaes MT, D'Souza S, Rizzo AA, Korza G, Walker GC, et al Interaction between the Rev1 C-Terminal Domain and the PolD3 Subunit of Polzeta Suggests a Mechanism of Polymerase Exchange upon Rev1/Polzeta-Dependent Translesion Synthesis. Biochemistry. 2016;55(13):2043–53. doi: 10.1021/acs.biochem.5b01282 ; PubMed Central PMCID: PMCPMC4898654. 26982350 10.1021/acs.biochem.5b01282 PMC4898654

[91] Pozhidaeva A, Pustovalova Y, D'Souza S, Bezsonova I, Walker GC, Korzhnev DM. NMR structure and dynamics of the C-terminal domain from human Rev1 and its complex with Rev1 interacting region of DNA polymerase eta. Biochemistry. 2012;51(27):5506–20. doi: 10.1021/bi300566z ; PubMed Central PMCID: PMCPMC3732116. 22691049 10.1021/bi300566z PMC3732116

[92] Sale JE, Batters C, Edmunds CE, Phillips LG, Simpson LJ, Szuts D. Timing matters: error-prone gap filling and translesion synthesis in immunoglobulin gene hypermutation. Philos Trans R Soc Lond B Biol Sci. 2009;364(1517):595–603. doi: 10.1098/rstb.2008.0197 ; PubMed Central PMCID: PMCPMC2660919. 19008194 10.1098/rstb.2008.0197 PMC2660919

[93] Waters LS, Walker GC. The critical mutagenic translesion DNA polymerase Rev1 is highly expressed during G(2)/M phase rather than S phase. Proc Natl Acad Sci U S A. 2006;103(24):8971–6. doi: 10.1073/pnas.0510167103 ; PubMed Central PMCID: PMCPMC1482550. 16751278 10.1073/pnas.0510167103 PMC1482550

[94] Minko IG, Harbut MB, Kozekov ID, Kozekova A, Jakobs PM, Olson SB, et al Role for DNA polymerase kappa in the processing of N2-N2-guanine interstrand cross-links. J Biol Chem. 2008;283(25):17075–82. doi: 10.1074/jbc.M801238200 ; PubMed Central PMCID: PMCPMC2427349. 18434313 10.1074/jbc.M801238200 PMC2427349

[95] Klug AR, Harbut MB, Lloyd RS, Minko IG. Replication bypass of N2-deoxyguanosine interstrand cross-links by human DNA polymerases eta and iota. Chem Res Toxicol. 2012;25(3):755–62. doi: 10.1021/tx300011w ; PubMed Central PMCID: PMCPMC3723381. 22332732 10.1021/tx300011w PMC3723381

[96] Ho TV, Guainazzi A, Derkunt SB, Enoiu M, Scharer OD. Structure-dependent bypass of DNA interstrand crosslinks by translesion synthesis polymerases. Nucleic Acids Res. 2011;39(17):7455–64. doi: 10.1093/nar/gkr448 ; PubMed Central PMCID: PMCPMC3177197. 21666254 10.1093/nar/gkr448 PMC3177197

[97] Raschle M, Knipscheer P, Enoiu M, Angelov T, Sun J, Griffith JD, et al Mechanism of replication-coupled DNA interstrand crosslink repair. Cell. 2008;134(6):969–80. doi: 10.1016/j.cell.2008.08.030 ; PubMed Central PMCID: PMCPMC2748255. 18805090 10.1016/j.cell.2008.08.030 PMC2748255

[98] Clauson C, Scharer OD, Niedernhofer L. Advances in understanding the complex mechanisms of DNA interstrand cross-link repair. Cold Spring Harb Perspect Biol. 2013;5(10):a012732 doi: 10.1101/cshperspect.a012732 ; PubMed Central PMCID: PMCPMC4123742. 24086043 10.1101/cshperspect.a012732 PMC4123742

[99] Scharer OD. Nucleotide excision repair in eukaryotes. Cold Spring Harb Perspect Biol. 2013;5(10):a012609 doi: 10.1101/cshperspect.a012609 ; PubMed Central PMCID: PMCPMC3783044. 24086042 10.1101/cshperspect.a012609 PMC3783044

[100] Krokan HE, Bjoras M. Base excision repair. Cold Spring Harb Perspect Biol. 2013;5(4):a012583 doi: 10.1101/cshperspect.a012583 ; PubMed Central PMCID: PMCPMC3683898. 23545420 10.1101/cshperspect.a012583 PMC3683898

[101] Sakofsky CJ, Ayyar S, Deem AK, Chung WH, Ira G, Malkova A. Translesion Polymerases Drive Microhomology-Mediated Break-Induced Replication Leading to Complex Chromosomal Rearrangements. Mol Cell. 2015;60(6):860–72. Epub 2015/12/17. doi: 10.1016/j.molcel.2015.10.041 ; PubMed Central PMCID: PMC4688117. 26669261 10.1016/j.molcel.2015.10.041 PMC4688117

[102] Kawamoto T, Araki K, Sonoda E, Yamashita YM, Harada K, Kikuchi K, et al Dual roles for DNA polymerase eta in homologous DNA recombination and translesion DNA synthesis. Mol Cell. 2005;20(5):793–9. Epub 2005/12/13. doi: 10.1016/j.molcel.2005.10.016 . 16337602 10.1016/j.molcel.2005.10.016

[103] Sarkies P, Reams C, Simpson LJ, Sale JE. Epigenetic instability due to defective replication of structured DNA. Mol Cell. 2010;40(5):703–13. Epub 2010/12/15. doi: 10.1016/j.molcel.2010.11.009 ; PubMed Central PMCID: PMC3145961. 21145480 10.1016/j.molcel.2010.11.009 PMC3145961

[104] Boersma V, Moatti N, Segura-Bayona S, Peuscher MH, van der Torre J, Wevers BA, et al MAD2L2 controls DNA repair at telomeres and DNA breaks by inhibiting 5' end resection. Nature. 2015;521(7553):537–40. Epub 2015/03/25. doi: 10.1038/nature14216 ; PubMed Central PMCID: PMC4481296. 25799990 10.1038/nature14216 PMC4481296

[105] Chun AC, Kok KH, Jin DY. REV7 is required for anaphase-promoting complex-dependent ubiquitination and degradation of translesion DNA polymerase REV1. Cell Cycle. 2013;12(2):365–78. doi: 10.4161/cc.23214 ; PubMed Central PMCID: PMCPMC3575465. 23287467 10.4161/cc.23214 PMC3575465

[106] Doles J, Oliver TG, Cameron ER, Hsu G, Jacks T, Walker GC, et al Suppression of Rev3, the catalytic subunit of Pol{zeta}, sensitizes drug-resistant lung tumors to chemotherapy. Proc Natl Acad Sci U S A. 2010;107(48):20786–91. doi: 10.1073/pnas.1011409107 ; PubMed Central PMCID: PMCPMC2996428. 21068376 10.1073/pnas.1011409107 PMC2996428

[107] Xie K, Doles J, Hemann MT, Walker GC. Error-prone translesion synthesis mediates acquired chemoresistance. Proc Natl Acad Sci U S A. 2010;107(48):20792–7. doi: 10.1073/pnas.1011412107 ; PubMed Central PMCID: PMCPMC2996453. 21068378 10.1073/pnas.1011412107 PMC2996453

[108] Xu X, Xie K, Zhang XQ, Pridgen EM, Park GY, Cui DS, et al Enhancing tumor cell response to chemotherapy through nanoparticle-mediated codelivery of siRNA and cisplatin prodrug. Proc Natl Acad Sci U S A. 2013;110(46):18638–43. doi: 10.1073/pnas.1303958110 ; PubMed Central PMCID: PMCPMC3832000. 24167294 10.1073/pnas.1303958110 PMC3832000

[109] Yang L, Shi T, Liu F, Ren C, Wang Z, Li Y, et al REV3L, a promising target in regulating the chemosensitivity of cervical cancer cells. PLoS ONE. 2015;10(3):e0120334 Epub 2015/03/18. doi: 10.1371/journal.pone.0120334 ; PubMed Central PMCID: PMC4364373. 25781640 10.1371/journal.pone.0120334 PMC4364373

[110] Wang W, Sheng W, Yu C, Cao J, Zhou J, Wu J, et al REV3L modulates cisplatin sensitivity of non-small cell lung cancer H1299 cells. Oncology reports. 2015;34(3):1460–8. Epub 2015/07/15. doi: 10.3892/or.2015.4121 . 26165320 10.3892/or.2015.4121

[111] Bartz SR, Zhang Z, Burchard J, Imakura M, Martin M, Palmieri A, et al Small interfering RNA screens reveal enhanced cisplatin cytotoxicity in tumor cells having both BRCA network and TP53 disruptions. Mol Cell Biol. 2006;26(24):9377–86. Epub 2006/09/27. doi: 10.1128/MCB.01229-06 ; PubMed Central PMCID: PMC1698535. 17000754 10.1128/MCB.01229-06 PMC1698535

[112] Lord CJ, Tutt AN, Ashworth A. Synthetic lethality and cancer therapy: lessons learned from the development of PARP inhibitors. Annual review of medicine. 2015;66:455–70. Epub 2014/10/24. doi: 10.1146/annurev-med-050913-022545 . 25341009 10.1146/annurev-med-050913-022545

[113] Kotov IN, Siebring-van Olst E, Knobel PA, van der Meulen-Muileman IH, Felley-Bosco E, van Beusechem VW, et al Whole genome RNAi screens reveal a critical role of REV3 in coping with replication stress. Molecular oncology. 2014;8(8):1747–59. Epub 2014/08/13. doi: 10.1016/j.molonc.2014.07.008 . 25113059 10.1016/j.molonc.2014.07.008 PMC5528584

[114] Mohni KN, Thompson PS, Luzwick JW, Glick GG, Pendleton CS, Lehmann BD, et al A Synthetic Lethal Screen Identifies DNA Repair Pathways that Sensitize Cancer Cells to Combined ATR Inhibition and Cisplatin Treatments. PLoS ONE. 2015;10(5):e0125482 Epub 2015/05/13. doi: 10.1371/journal.pone.0125482 ; PubMed Central PMCID: PMC4428765. 25965342 10.1371/journal.pone.0125482 PMC4428765

[115] Sonoda E, Okada T, Zhao GY, Tateishi S, Araki K, Yamaizumi M, et al Multiple roles of Rev3, the catalytic subunit of polzeta in maintaining genome stability in vertebrates. EMBO J. 2003;22(12):3188–97. Epub 2003/06/14. doi: 10.1093/emboj/cdg308 ; PubMed Central PMCID: PMC162160. 12805232 10.1093/emboj/cdg308 PMC162160

[116] Yamanaka K, Dorjsuren D, Eoff RL, Egli M, Maloney DJ, Jadhav A, et al A comprehensive strategy to discover inhibitors of the translesion synthesis DNA polymerase kappa. PLoS ONE. 2012;7(10):e45032 Epub 2012/10/12. doi: 10.1371/journal.pone.0045032 ; PubMed Central PMCID: PMC3466269. 23056190 10.1371/journal.pone.0045032 PMC3466269

[117] Mizushina Y, Motoshima H, Yamaguchi Y, Takeuchi T, Hirano K, Sugawara F, et al 3-O-methylfunicone, a selective inhibitor of mammalian Y-family DNA polymerases from an Australian sea salt fungal strain. Marine drugs. 2009;7(4):624–39. Epub 2010/01/26. doi: 10.3390/md7040624 ; PubMed Central PMCID: PMC2810227. 20098603 10.3390/md7040624 PMC2810227

[118] Actis ML, Ambaye ND, Evison BJ, Shao Y, Vanarotti M, Inoue A, et al Identification of the first small-molecule inhibitor of the REV7 DNA repair protein interaction. Bioorganic & medicinal chemistry. 2016;24(18):4339–46. Epub 2016/07/28. doi: 10.1016/j.bmc.2016.07.026 . 27448776 10.1016/j.bmc.2016.07.026 PMC5688848

